# Salivary Stress-Related Responses in Tinnitus: A Preliminary Study in Young Male Subjects with Tinnitus

**DOI:** 10.3389/fnins.2016.00338

**Published:** 2016-07-20

**Authors:** Ola A. Alsalman, Denise Tucker, Sven Vanneste

**Affiliations:** ^1^Lab for Clinical and Integrative Neuroscience, School of Behavioral and Brain Sciences, The University of Texas at DallasDallas, TX, USA; ^2^Department of Communication Sciences and Disorders, University of North Carolina at GreensboroGreensboro, NC, USA

**Keywords:** tinnitus, stress-related responses, salivary alpha amylase, salivary cortisol, salivary neopterin

## Abstract

**Objective:** This preliminary study examined if baseline measures of stress-related biomarkers as measured by salivary secretions of specific autonomic [measured by salivary α-amylase (sAA)], endocrine (measured by salivary cortisol), and immune (measured by salivary neopterin) responses are greater in male subjects with tinnitus in response to an induced-stress task.

**Method:** Twenty male subjects with no significant hearing loss, 10 with tinnitus, and 10 without tinnitus were enrolled in this study.Salivary secretions were collected before and after the induced stress task at four different time intervals.

**Results:** sAA levels were lower in the tinnitus group in comparison to subjects without tinnitus, suggesting impaired sympathetic activity in the subjects with tinnitus although these levels remained stable throughout the stress experiment.While no significant effects could be obtained for salivary cortisol or neopterin, salivary neopterin levels were trending toward significance over all measurements. Behavioral measures of stress were found to correlate negatively with measures of sAA and salivary neopterin.

**Conclusion:** The results of this study suggest impaired stress-related sAA mechanisms in male subjects with tinnitus, as evidenced by the different stress reactions induced in the endocrine system (as measured by salivary cortisol) and the immune system (as measured by salivary neopterin).

## Introduction

Tinnitus is a sound that is perceived in the absence of an external acoustic stimulus (Jastreboff and Hazell, 1993). The complexity in identifying the underlying mechanism of tinnitus generation is in part related to the subjective nature of this condition, along with the cascade of associated emotional and psychological reactions accompanying its occurrence. Patients with chronic tinnitus can experience a disabling sense of depression, anxiety, stress, and sleep difficulties (Halford and Anderson, 1991; Folmer et al., 1999; Folmer and Griest, 2000; Shargorodsky et al., 2010).

Stress is widely acknowledged as a predisposing and precipitating factor in different neurological and psychiatric illnesses. While acute stress promotes adaptation, prolonged stress over time leads to wear-and-tear on the body—so called the allostatic load, which comprises the negative physiological and psychological effects of stress. Stress induces secretion of corticotropin-releasing hormone from the hypothalamus, which stimulates the secretion of adrenocorticotropin from the pituitary gland. That release of adrenocorticotropin to blood causes the adrenal gland to secrete stress hormones, such as cortisol (Hebert and Lupien, 2007). In addition, several studies have indicated that the sympathetic nervous system (SNS) plays an important role in stress (Cohen and Khalaila, 2014; Lupis et al., 2014; McKune et al., 2014; Carnuta et al., 2015; Het et al., 2015). Given that secretions from salivary glands are modulated by direct sympathetic innervations, salivary α-amylase (sAA) is proposed to be a surrogate marker of SNS activity, with changes in sAA reflecting sympathetic influences on salivary glands.

Reaction to acute stress is not only limited to activity that involves the activation of sympathetic and endocrine systems, but can also be observed in the immune system (Fuchs et al., 1993a; Moynihan, 2003). In the course of an immune response, a whole spectrum of active substances is generated, one of them being neopterin. Neopterin is released into the body by the kidneys and produced by monocytes/macrophages after stimulation by the cytokine interferon-γ (Widner et al., 2002). Both animal and human research has demonstrated that an acute stressor does change neopterin levels (Breinekova et al., 2007; Lindsay et al., 2015). Particularly, stress is found to impact hormonal regulatory function, causing immune cells to become insensitive to hormones and consequently resulting in suppression of immune system responses, triggering increased susceptibility to diseases (Cohen et al., 1991; van West and Maes, 2001).

Taken as a whole, alteration of individual physiological status as a result of a stressor is generally accepted as an underlying mechanism for stress generation (Chrousos and Gold, 1992; Chrousos, 2000; Tsigos and Chrousos, 2002; Kudielka and Wust, 2010). This mechanism involves activation of both the hypothalamic-pituitary-axis (HPA) and SNS. Given that there is a clear association between stress and tinnitus (Hinton et al., 2006; Shargorodsky et al., 2010; Nondahl et al., 2011; Kim et al., 2015), similar HPA and SNS salivary reactions can be expected in subjects with tinnitus.

With this in mind, the aim of this preliminary study is to examine autonomic, immune, and endocrine stress-related markers in male subjects with tinnitus in comparison to healthy controls (i.e., subjects without tinnitus). We wanted to examine if the concentrations of circulating sAA and salivary cortisol in male subjects with tinnitus differ from healthy controls. In addition, since increase in immune system function can lead to deregulation of the endocrine and immune systems, we also looked at salivary neopterin as an immune system marker in relation to tinnitus. As such, we hypothesized that salivary stress related markers could be used to distinguish stress influences and variations between the two groups.

## Materials and methods

### Participants

Male subjects were invited to participate in a research study by posting flyers at the University of North Carolina at Greensboro (UNCG) campus and surrounding areas. Subjects were excluded if they were smokers, had any dental work within the 48 h prior to sample collection, were prescribed or currently taking any medications for depression, anxiety, stress, bipolar disorder, epilepsy, thyroid dysfunction, schizophrenia, or insomnia, or any other neurological diseases. Because of monthly hormonal fluctuation (specifically cortisol) due to the menstrual cycle, only males were included in this study. In addition, due to the hormonal changes, which accompany increased age, the age limits were set from 18 to 35 years.

In total, 16 male subjects with tinnitus and 20 male subjects without tinnitus applied to participate in the study. However, 6 male subjects with tinnitus and 10 male subjects without tinnitus were excluded because they did not fit the inclusion criteria. Twenty male subjects with no significant hearing loss, 10 with tinnitus (mean age: 23.9 years; *Sd* = 3.78) and 10 without tinnitus (mean age: 23.6 years; *Sd* = 4.47), were enrolled in this study. All research involved in this study was approved by the authors' Institutional Review Board (The university of North Carolina at Greensboro IRB #12-0223), and all subjects gave signed informed consent prior to enrollment. All clinical investigation was conducted according to the principles expressed in the Declaration of Helsinki. Informed consent, written or oral, was obtained from the participants.

### Questionnaires and audiometric testing

Male subjects with tinnitus completed all questionnaires, while those without tinnitus only filled in four out of the six questionnaires (tinnitus medical history intake questionnaire and tinnitus severity index excluded). All questionnaires were mailed out to subjects prior to their scheduled lab visit. In addition, audiometric testing was administrated to determine subjects hearing level. Subjects with tinnitus had their tinnitus pitch and loudness match measured.

### Stress test

To induce stress, we adapted the mental arithmetic task, part of the the Trier Social Stress Test (TSST), which has proven to be effective in inducing physiological changes in response to stress (Kirschbaum et al., 1993). After collecting the baseline measure, subjects were asked to perfrom a mental arthmetic task, by counting backwards, starting at 5000, in intervals of seven, for up to 5 min.

### Clinical assessment

The neuromeric rating scale (NRS) and saliva collections were assessed before beginning the stress task as well as 5, 30, and 60 min after inducing stress.

#### Numeric rating scale (NRS) of acute stress

Numeric Rating Scale (NRS) of acute stress was assessed subjectively by asking subjects to rate how stressed they were before collecting each of the four saliva samples on a scale from 0 (= no stress) to 10 (= extremely stressed).

#### Saliva collection

Commercial collection aids were used from the Salimetrics laboratory (Salimetrics, LLC, USA). All samples were measured using a kinetic kit. The collection aid was used to collect unstimulated saliva samples via a passive drool method. All saliva samples obtained were stored in a 2 ml cryovials, and immediately stored in an −80°C laboratory freezer. Collected saliva sample were de-identified, and subjects were assigned a number that was used in the saliva testing and in subsequent psychometric scores analysis. Prior to saliva collections, all subjects were instructed to avoid food, dental surgery, sugary drinks, alcohol, caffeine, nicotine, acidic drinks, and excessive napping or exercising on scheduled lab visits, for at least an hour before collecting the saliva samples. In addition, subjects' were instructed not to brush their teeth within 45 min prior to sample collection in order to avoid any risk of lowering pH levels and influencing bacterial growth. Due to the sensitive nature of some of the targeted biomarkers and to minimize possible influence of circadian rhythm, all collection procedures took place at the same time of day: 6:00 p.m.

#### Flow rate correction

If correction for saliva flow rate is not made, there will be variation in the concentration per volume unit from subject-to-subject. This variation could cause problems in statistical analysis that might make it difficult to reveal a biomarker-behavior-relationship.

The correction method explained below enabled us to correct for sAA concentration per minute for each trial result:

(1) Flow rate(ml/min)=((saliva(ml)/(time(min)))

There was no need to correct sAA activity for flow rate since the alpha amylase levels were not correlated with flow rate at each measurement.

#### Saliva processing

Upon completion of the collection procedures, a total of 80 saliva samples were packed in dry ice and sent to the Salimetrics laboratory (Salimetrics, LLC, USA) for analysis. Salivary cortisol and neopterin were measured in duplicates, while sAA was measured in singulate.

### Statistics

All parameters, along with each subject's demographic and psychometric data, were analyzed using descriptive statistics. A Mann–Whitney test was conducted to determine whether there was a difference in the Pittsburgh sleep quality index (PSQI) and the perceived stress scale (PSS) between subjects, with and without tinnitus. In addition, Spearman's Rho correlation was computed to determine associations between baseline measures of PSS and NRS.

To test if there is a significant difference between sAA, salivary cortisol and salivary neopterin between subjects, with and without tinnitus at baseline, a Mann–Whitney test was conducted. In addition Spearman's Rho correlation was computed to determine associations between PSS and baseline measures of sAA, salivary cortisol, and salivary neopterin, respectively.

A Kruskal–Wallis test was applied using the values of the different time intervals (baseline and post-test) as the test variables, and the groups, control vs. tinnitus, as the grouping variables, for each of the three stress-related markers (sAA, salivary cortisol, and salivary neopterin) to examine any significant effect. When significance value was obtained a follow-up with Mann–Whitney test was conducted. To avoid type I error a Bonferroni correction was made adjusting the *p*-value by dividing 0.05/(# comparison). Effect size was computed manually using the following formula:

(2) $$r=Z/\sqrt{\left(N\right)}$$

Similarly, a Kruskal–Wallis test was conducted to examine any evidence of difference on each of the three biomarkers for the follow-up measures at 30 and 60 min. When significance value was obtained a follow-up with Mann–Whitney test was conducted.

We applied a Mann–Whitney test to determine whether there was a difference in flow rate measures for sAA at baseline, 5, 30, and 60 min post-test. In addition, Spearman's Rho was conducted between flow rate and sAA at baseline, and 5, 30, and 60 min.

To confirm the experimental validation of the stress test we applied a Kruskal–Wallis test using values of NRS of the different time intervals as the test variables, and the groups, control vs. tinnitus, as the grouping variables. A similar analysis was applied for post-measurements.

To look for the association or dissociation between sAA and salivary cortisol, we calculated area under the curve (AUC) (Pruessner et al., 2003; Ali and Pruessner, 2012). AUC, with respect to ground, was first calculated for sAA, cortisol, and neopterin using the trapezoid formula described previously (Pruessner et al., 2003):

$$\begin{array}{l} AUC=\sum_{i=1}^{n-1}\frac{\left(m_{\left(i+1\right)}+m_{i}\right)\cdot t_{i}}{2}\\ \;m\;=\text{ measurements}\\ \;t=\text{ denoting the distance }(\text{time})\text{ between the measurements} \end{array}$$

This score incorporates information regarding both baseline and reactivity individually for sAA and cortisol within one score. We then divided the AUC of sAA by the AUC of salivary cortisol to derive an overall ratio (Ali and Pruessner, 2012), representing the variable of amylase over cortisol based on the individual AUC variables, which we named AOC. This variable can be thought of as the variation in sAA levels after correcting for variations in salivary cortisol. Complimentarily, the ratio variable cortisol over amylase (COA) was calculated by dividing the AUC of salivary cortisol by the AUC of sAA. This variable can be thought of as the variation of cortisol corrected for the variation in amylase. We also divided the AUC of sAA and the AUC of salivary neopterin to derive an overall ratio variable of amylase over neopterin (AON) as well as the AUC of salivary neopterin by the AUC of sAA (NOA). In addition, we also divided the AUC of salivary cortisol and the AUC of salivary neopterin (CON) as well as the AUC of salivary neopterin by the AUC of cortisol (NOC). These various ratio computations are based on Ali and Pruessner's work on assessing sAA over cortisol ratio as a marker to assess deregulation of the stress system (Ali and Pruessner, 2012).

AUC of sAA, AUC of salivary cortisol, alpha amylase over cortisol (AOC), cortisol over alpha amylase (COA), alpha amylase over neopterin (AON), neopterin over alpha amylase (NOA), cortisol over neopterin (CON), and neopterin over cortisol (NOC) were then z-transformed to standardized measurements.

A Mann–Whitney test was applied to evaluate differences of AUC of sAA, AUC of salivary cortisol, and AUC of salivary neopterin between subjects with and without tinnitus. Spearman's Rho were computed between AUC of salivary cortisol and AUC of salivary neopterin and AUC of sAA and AUC of salivary cortisol.

A logistic regression including AUC of sAA, AUC of salivary cortisol and AUC of salivary neopterin as independent variables and group (subjects with tinnitus vs. subjects without tinnitus) as dependent variables was also conducted.

Spearman's correlations were conducted to examine the associations between the standardized AUC of sAA, AUC of salivary cortisol, α-amylase over cortisol, cortisol over α-amylase, α-amylase over neopterin, neopterin over α-amylase, cortisol over neopterin, and neopterin over cortisol with subjective indexes of stress. A 5% level of significance and a Spearman's Rho two-tailed analyses were adopted for all analyses. All statistical analyses were completed using the SPSS software package 22.

## Results

### Descriptive characteristics

#### Overall subjects

Both subjects with tinnitus and subjects without tinnitus reported to have no serious health conditions, which required immediate or continuous medical attention. When given the choice to rate their overall health, 60% of subjects without tinnitus described their health as “excellent.” The majority of subjects with tinnitus (80%) indicated their overall health as “good” or “excellent.” Four subjects with tinnitus experienced one to three headaches per week in the month prior to experimentation; however, 80% reported these headaches not to be a significant problem. Exposure to noise was reported as one out of the two main causes of tinnitus. The remaining subjects reported exposure to stressful events as the primary cause of their tinnitus.

A Mann–Whitney test indicated that the PSQI scores were significantly greater for subjects with tinnitus (*M* = 4.9, *Sd* = 2.18, *Md* = 5.50) than subjects without tinnitus (*M* = 2.70, *Sd* = 1.05, *Md* = 2), *U* = 20.50, *p* = 0.02, *r* = 0.51. Similarly, the PSS score for subjects with tinnitus (*M* = 20.30, *Sd* = 6.86, *Md* = 20) were greater than subjects without tinnitus (*M* = 10.10, *Sd* = 2.80, *Md* = 10), *U* = 6.50, *p* = 0.001, *r* = 0.73. Pure tone audiometry results indicated that all subjects had normal hearing thresholds of 20 dB HL or better (with tinnitus, right ear *M* = 16, *Sd* = 10.72; without tinnitus, right ear *M* = 19.66, *Sd* = 12.24) and (with tinnitus, left ear *M* = 22, *Sd* = 9.30; without tinnitus, left ear *M* = 14.50, *Sd* = 10.20). A comparison between the pure tone averages revealed no significant effect between groups. No significant difference between groups was revealed for the pure tone averages.

#### Tinnitus characteristics

Five tinnitus subjects reported to have a gradual onset of tinnitus, while five subjects reported to have a sudden onset. The average duration of tinnitus was 3.30 years (*Sd* = 1.63). Six subjects perceived their tinnitus bilaterally, while four perceived it unilaterally. The average pitch match for both the left and right ears were approximately 3 kHz (right ear *M* = 3525, *Sd* = 2180; left ear *M* = 3050, *Sd* = 1978), while average loudness match ranged from 13 to 58 dB HL. The range of loudness match varied from 13 to 44 dB HL in the right ear and 13 to 58 dB HL in the left ear (right ear *M* = 30.50, *Sd* = 11.24; left ear *M* = 31.20, *Sd* = 13.33).

According to the TSI scores (*M* = 19.20, *Sd* = 6.90), tinnitus was not considered to be a bothersome or debilitating problem for subjects with tinnitus. Pure tone ringing was the most common tinnitus sound described by the tinnitus subjects (70%), while hissing and music were the least described tinnitus sounds (20%); 10% reported their tinnitus to be a combination of different tones.

#### Baseline measures

**Figure 1:**
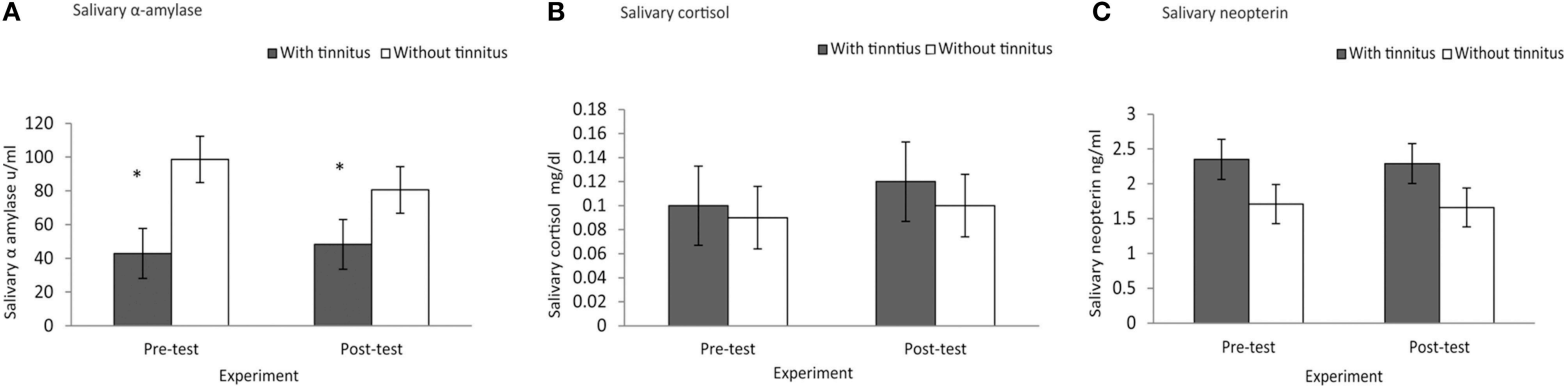
**Experimental effect of group differences at baseline vs.post-test for (A) salivary α amylase, (B) salivary cortisol, and (C) salivary neopterin. (A)** Subjects with tinnitus (*M* = 42.84, *Sd* = 24.38) had lessened salivary α amylase scores than those without tinnitus (*M* = 98.60, *Sd* = 61.51). **(A)** *Indicates significant differences. **(B)** Salivary cortisol, and **(C)** salivary neopterin yielded no statistical significant differences between baseline and post-test measure.

A Mann–Whitney test revealed significant difference for baseline sAA (μ/ml) which demonstrated lower score for subjects with tinnitus (*M* = 42.84, *Sd* = 24.38, *Md* = 42.65) than subjects without tinnitus (*M* = 98.60, *Sd* = 61.51, *Md* = 65.45), *U* = 20, *p* = 0.023, *r* = −0.50. Baseline concentrations of salivary cortisol (mg/dl) (subjects with tinnitus *M* = 0.10, *Sd* = 0.09, *Md* = 0.07; subjects without tinnitus *M* = 0.09, *Sd* = 0.06, *Md* = 0.09), *U* = 49, *p* = 0.94, *r* = 0.01, yielded no statistical significant difference. Similarly, salivary neopterin (ng/ml) yielded no significant difference (subjects with tinnitus *M* = 2.35, *Sd* = 0.75, *Md* = 2.54; subjects without tinnitus *M* = 1.71, *Sd* = 0.99, *Md* = 1.79), *U* = 28, *p* = 0.09, *r* = 0.37. See Figures 1A–C.

**Figure 2:**
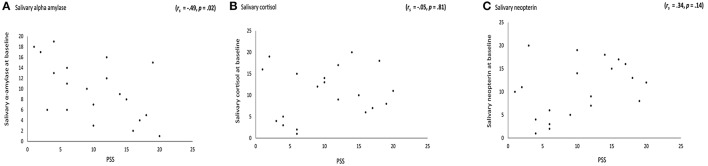
**Correlation between the perceived stress scale (PSS) and stress-related markers**. **(A)** A significant negative correlation (*r*_*s*_ = −0.49 *p* = 0.02) between PSS and baseline measures of salivary α amylase, indicating the higher the stress the lower salivary α amylase concentrations. **(B)** No correlation between PSS scores and baseline measures of salivary cortisol was obtained (*r*_*s*_ = −0.05 *p* = 0.81). Similarly, **(C)** PSS with baseline measures of salivary neopterin (*r*_*s*_ = 0.34 *p* = 0.14), yielded no statistical significance.

There was a significant correlation between scores the PSS and baseline measures of sAA (μ/ml) (*r*_*s*_ = −0.49 *p* = 0.02), indicating that higher stress rates corresponded to lower concentrations of sAA (see Figure 2A). The correlation between the related-stress psychometric scores of the PSS and baseline measures of salivary cortisol (mg/dl) (*r*_*s*_ = −0.05, *p* = 0.81) and salivary neopterin (ng/ml) (*r*_*s*_ = 0.34, *p* = 0.14) yielded no statistical significance. See Figures 2B,C.

### Experiment

#### Stress test

A Kruskal–Wallis test was conducted to determine whether baseline salivary stress-related measures of subjects with tinnitus and subjects without tinnitus varied from post-test measure. The test, which was corrected for tied ranks, showed that in regard to sAA (μ/ml) (*x*^2^ = 5.14, *p* = 0.02) results yielded a significant effect from post-test measure. Specifically, baseline measures of sAA for subjects with tinnitus were significantly lower (*M* = 42.84, *Sd* = 24.38, *Md* = 42.65) than subjects without tinnitus (*M* = 98.60, *Sd* = 61.51, *Md* = 65.45), *U* = 20, *p* = 0.02, *r* = −0.50.

No statistical significant difference was found for salivary cortisol (mg/dl) at baseline (*x*^2^ = 0.006, *p* = 0.09), and post-test measure (*x*^2^ = 0.07, *p* = 0.79). Similarly, there was no statistical significance difference demonstrated for salivary neopterin (ng/ml) at baseline (*x*^2^ = 2.76, *p* = 0.09), and post-test measure (*x*^2^ = 1.75, *p* = 0.18).

#### Follow-up

**Figure 3:**
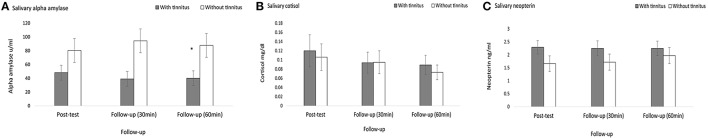
**Follow-up effect of group differences at 5, 30, and 60 min post-test measures. (A)** Salivary α amylase yielded no statistical significant difference for 30 min post-test, however a significant difference was obtained for 60 min post-test Specifically, subjects with tinnitus (*M* = 40.21, *Sd* = 30.06) had lower measures of salivary α-amylase than subjects without tinnitus (*M* = 87.81, *Sd* = 48.04). **(B)** Salivary cortisol and **(C)** salivary neopterin yielded no statistical significant difference for the effect of time. *Indicates significant difference.

With regard to sAA (μ/ml) there was no statistical significant difference between post-test measure and 30 min fellow-up (*x*^2^ = 4.80, *p* = 0.02). However, a significant difference was obtained at 60 min follow-up (*x*^2^ = 7, *p* = 0.008). Specifically, subjects with tinnitus (*M* = 40.21, *Sd* = 30.06, *Md* = 22.95) had lower measures of sAA than subjects without tinnitus (*M* = 87.81, *Sd* = 48.04, *Md* = 80.20), *U* = 15, *p* = 0.008, *r* = −0.59. See Figure 3A.

No statistical significant difference was demonstrated for salivary cortisol (mg/dl) at 30 min (*x*^2^ = 0.20, *p* = 0.65), and 60 min (*x*^2^ = 14, *p* = 0.70). Similarly, there was no significant difference demonstrated for salivary neopterin (ng/ml) at 30 min (*x*^2^ = 1.12, *p* = 0.29), and 60 min follow up (*x*^2^ = 1.28, *p* = 0.25). See Figures 3B,C.

#### Control of flow rate of salivary α-amylase

A Mann–Whitney test of sAA flow rate reveled no statistical significant difference of flow rate measures in (ml/min) at baseline (subjects with tinnitus *M* = 1.85, *Sd* = 0.85, *Md* = 1.50; subjects without tinnitus *M* = 2.02, *Sd* = 1.20, *Md* = 1.75), *U* = 47, *p* = 0.81, *r* = −0.05, at 5 min post-test measure (subjects with tinnitus *M* = 2.21, *Sd* = 0.78, *Md* = 2; subjects without tinnitus *M* = 1.79, *Sd* = 0.88, *Md* = 1.62), *U* = 30.50, *p* = 0.13, *r* = −0.30, at 30 min follow-up measure (subjects with tinnitus *M* = 2.02, *Sd* = 0.45, *Md* = 2; subjects without tinnitus *M* = 1.79, *Sd* = 0.84, *Md* = 1.50), *U* = 31, *p* = 0.13, *r* = −0.30, as well as at 60 min follow-up measure (subjects with tinnitus *M* = 1.77, *Sd* = 0.40, *Md* = 2; subjects without tinnitus *M* = 1.59, *Sd* = 0.80, *Md* = 1.20), *U* = 34, *p* = 0.21, *r* = −0.27. In addition, Spearman's Rho revealed no significant correlation between flow rate and sAA activity at baseline (*r*_*s*_ = −0.21, *p* = 0.35), 5 min (*r*_*s*_ = 0.08, *p* = 0.73), 30 min (*r*_*s*_ = 0.22, *p* = 0.34), and 60 min (*r*_*s*_ = −0.07, *p* = 0.75) post-test.

### Experimental validation (baseline vs. 5 min post-test)

**Figure 4:**
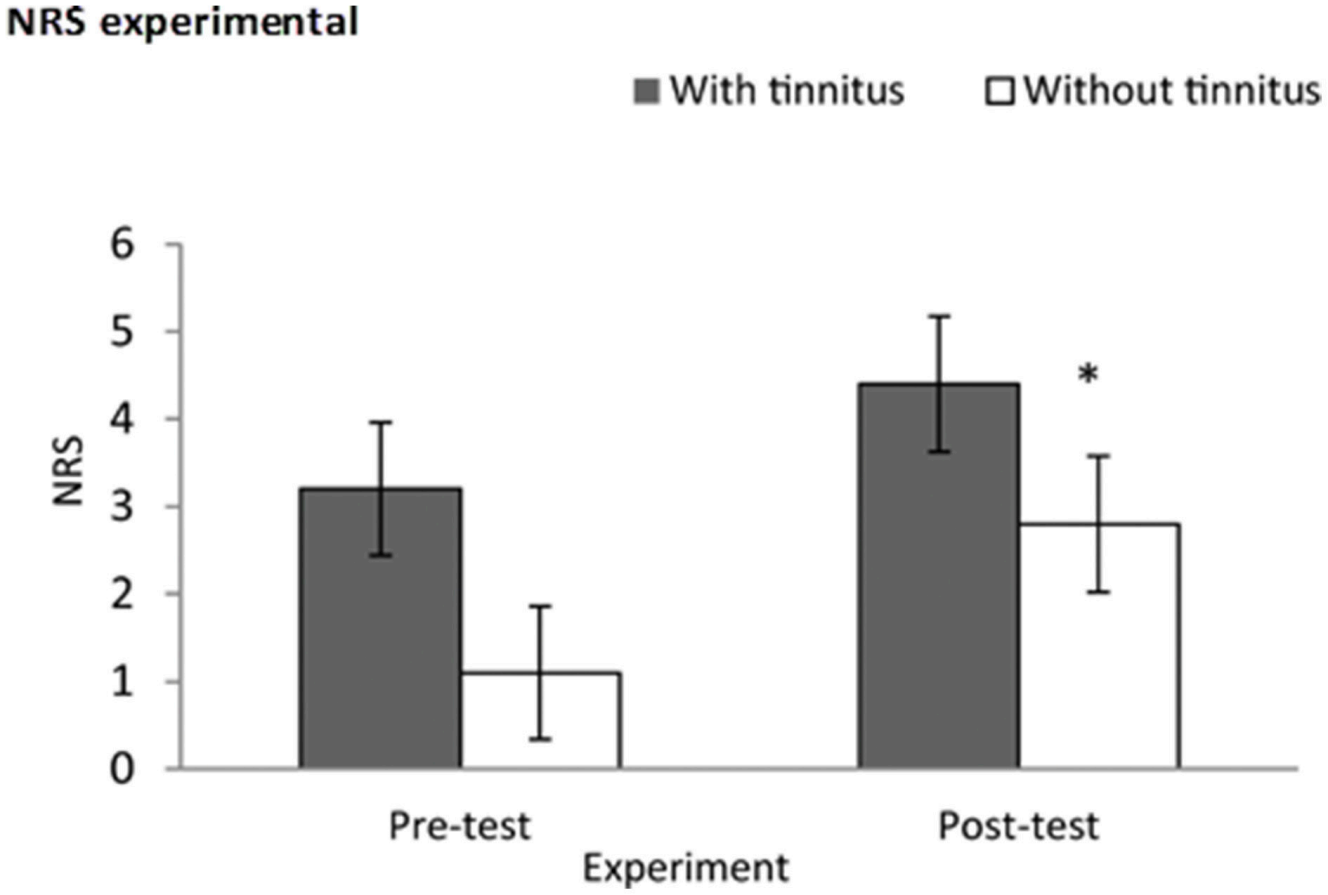
**Experimental validation using a numeric rating scale**. Subjects had increases stress scores post-test in comparison to baseline; this was characterized by subjects with tinnitus (*M* = 4.40, *Sd* = 2.50) exhibiting higher stress scores than subjects without tinnitus (*M* = 2.80, *Sd* = 2.39). *Indicates significant difference.

A Kruskal–Wallis test was conducted for NRS at baseline and 5 min post-test, and revealed statistical difference at the edge of significance (*x*^2^ = 3.69, *p* = 0.05), A Mann–Whitney test showed that subjects with tinnitus had a greater increase in NRS scores post-test (*M* = 4.40, *Sd* = 2.50, *Md* = 4.50) than subjects without tinnitus (*M* = 2.80, *Sd* = 2.39, *Md* = 2.50), *U* = 25, *p* = 0.05, *r* = −0.42. See Figure 4.

### Sensitivity analysis

A Mann–Whitney test revealed a significant effect for AUC of sAA. Subjects with tinnitus (*M* = 2510.20, *Sd* = 1566.34, *Md* = 2548.12) had lower scores than subjects without tinnitus (*M* = 5369.55, *Sd* = 2975.72, *Md* = 4317.75), *U* = 19, *p* = 0.01, *r* = −0.52. No significant effects were obtained for AUC of salivary cortisol, AUC of salivary neopterin.

Significant correlations were obtained between AUC of salivary cortisol and AUC of salivary neopterin (*r*_*s*_ = 0.53, *p* = 0.01). Between AUC of sAA and respectively AUC of salivary cortisol (*r*_*s*_ = 0.19, *p* = 0.40) and AUC of salivary neopterin (*r*_*s*_ = −0.22, *p* = 0.35) no significant correlation could be obtained.

**Figure 5:**
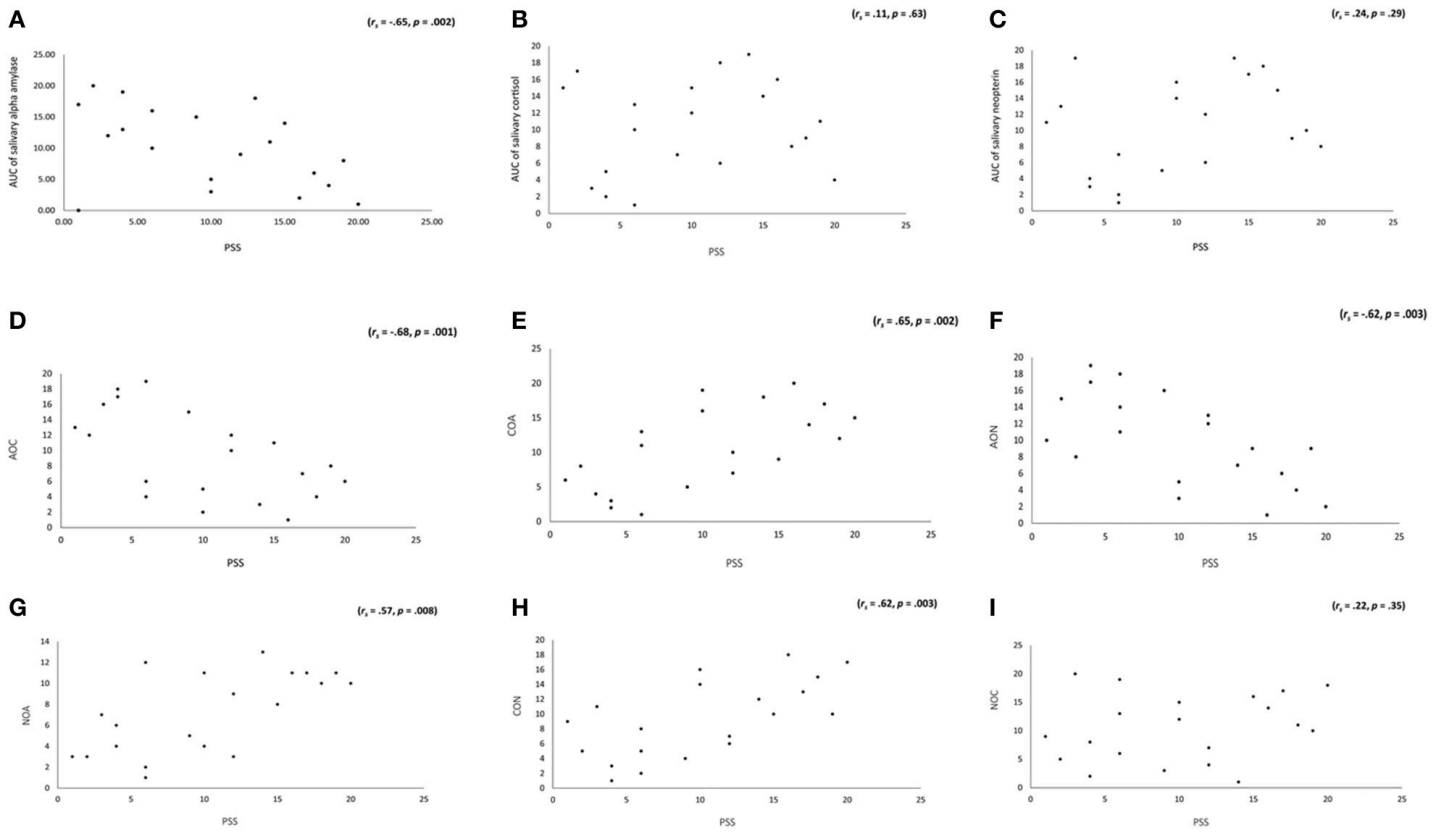
**(A)** Significant correlations was obtained between AUC of salivary α-amylase, but not with **(B)** AUC salivary cortisol, or **(C)** AUC neopterin.Spearman's Rho were significant for **(D)** AOC, **(E)** COA, **(F)** AON, **(G)** NOA, and **(H)** CON, but not for **(I)** NOC. This suggests that the higher the scores of the PSS the lower the individual scores of AUC for salivary α-amylase, salivary cortisol, and salivary neopterin, and the higher the likelihood that subjects has chronic stress.

A logistic regression including AUC of sAA, AUC of salivary cortisol and AUC of salivary neopterin as independent variables and group (subjects with tinnitus vs. subjects without tinnitus) as dependent variable was conducted. This analysis revealed an overall effect, c^2^ = 11.87, *p* = 0.008, Nagelkerke *R*^2^ = 0.62. A closer look indicated that AUC of sAA was a good predictor (*W* = 4.26, *p* = 0.04, β = −2.50) indicating that 84.2% of the subjects could be correctly classified. The β-value further indicated that the lower the individual score is on AUC of sAA the higher the probability is that a subject has tinnitus. Neither AUC of salivary cortisol nor AUC of salivary neopterin reached significance. In addition, a Spearman's correlation revealed a significant effect between PSS and respectively, AUC of sAA (*r*_*s*_ = −0.65, *p* = 0.002), α-amylase over cortisol (*r*_*s*_ = −0.68, *p* = 0.001), cortisol over α-amylase (*r*_*s*_ = 0.65, *p* = 0.002), α-amylase over neopterin (*r*_*s*_ = −0.62, *p* = 0.003), neopterin over α-amylase (*r*_*s*_ = 0.57, *p* = 0.008), and cortisol over neopterin (*r*_*s*_ = 0.62, *p* = 0.003), but not neopterin over cortisol (*r*_*s*_ = 0.22, *p* = 0.35). The correlations suggest that the higher the stress score, the lower the individual score on AUC of sAA, α-amylase over cortisol, or α-amylase over neopterin, indicating a higher probability that a subject has stress and vice versa. The higher an individual score is on cortisol over α-amylase, neopterin over α-amylase, and cortisol over neopterin, the higher the likelihood is that a subject has chronic stress or vice versa (as suggested by the positive correlation). No significant correlations were obtained for AUC of salivary cortisol (*r*_*s*_ = 0.11, *p* = 0.63), and AUC of salivary neopterin (*r*_*s*_ = 0.24, *p* = 0.29). For an overview, see Figure 5.

## Discussion

This is a preliminary study to examine the feasibility of utilizing stress related markers in tinnitus. The data from this preliminary study will help us pursue a larger investigation on the feasibility of using salivary stress related responses in tinnitus. To our knowledge, this is the first study with regard to the examination of sAA, salivary cortisol, and salivary neopterin, simultaneously, in male subjects with tinnitus. The results of this preliminary study show that sAA levels were lower in the tinnitus group in comparison to subjects without tinnitus, suggesting impaired sympathetic activity in the tinnitus group. Furthermore, sAA remained stable throughout the stress experiment (i.e., acute stress); further confirming impaired sympathetic activity in the tinnitus group. The results of this preliminary study found no correlations between sAA and salivary cortisol and salivary neopterin, respectively, highlighting the distinctiveness of sAA as a stress-related marker. Furthermore, sAA ratio was found to be more strongly associated with measures of stress and depression and is regarded as an index of deregulation in the stress system (Ali and Pruessner, 2012). In addition, this preliminary data found a strong effect observed for sAA in relation to baseline, stress test, follow-up and AUC, indicating that the expected value of sAA response decreases as stress increases, particularly for the tinnitus group.

### Salivary α-amylase, cortisol, and neopterin

An increasing amount of research is supporting the proposal of a dynamic relationship between tinnitus and stress (Vanneste et al., 2010; De Ridder et al., 2011). Stress induces biological changes, such as alterations in stress-related markers. sAA was suggested as a valuable index for the assessment of pain (Shirasaki et al., 2007; Ahmadi-Motamayel et al., 2013; Ferrara et al., 2013; Liu et al., 2013), psychological stress (Chatterton et al., 1997) and physical stress (Chatterton et al., 1996). Thus, because of the symptomatic similarities between tinnitus, stress, and pain, it may be expected that a common pathophysiology may underlie these conditions. There are reports of lower sAA in chronically stressed patients with mood depressive disorders (Cubala and Landowski, 2014) and personality disorders (Nater et al., 2010). It is known that chronic activation of this stress system can lead to changes in functioning (i.e., allostatic load), leading to impaired or inadequate responses to subsequent challenges (Chrousos, 2009). This can be confirmed by our data. During a stress test, no changes could be obtained in the tinnitus group; in the healthy controls, immediate changes were observed. Previous research suggests that patients habituated to stress are indicated by a decrease or decline of sAA concentrations (Schumacher et al., 2013, 2014). Interestingly, previous research in tinnitus has revealed that highly distressed tinnitus patients show increased activity in specific brain areas (i.e., insula, dorsal, and subgenual anterior cingulate cortex; Vanneste et al., 2010, 2014) that are known to be mediated by the SNS (Vanneste and De Ridder, 2013).

With regard to salivary cortisol, no effect was found in relation to stress between the two groups at baseline. However, a significant positive relationship was obtained for salivary cortisol after correcting for salivary neopterin with stress independent of whether the subject had tinnitus or not. Our finding is in line with findings of a recent report showing no differences in responses of the HPA axis, as measured by salivary cortisol after exposure to stressful events (McKune et al., 2014). This could be indicative of the effect the type of stress (i.e., acute vs. chronic) has on the activation of the HPA axis. Taking into consideration that salivary cortisol is influenced by circadian rhythm (Lac, 2001), our finding can be considered a reflection of cortisol systematic influence by the circadian rhythm, with concentrations being higher during the day and lower in the evening.

A key characteristic of HPA functioning that has been frequently observed is the habituation after repeated exposure to an initially stressful event (i.e., tinnitus) (Wust et al., 2005). Hebert and Lupien observed that basal levels of salivary cortisol are chronically changed in tinnitus patients (Hebert et al., 2004); however, they also report a blunted cortisol response to acute stress in patients who had experienced tinnitus for longer duration (Hebert and Lupien, 2007). Studies document that a habituation of adrenocortical responses does not necessarily occur (Al'Absi et al., 1997). It seems that characteristics of the stressor are important mediators determining the development of habituation, including intensity (Marti et al., 2001) and frequency (Ma and Lightman, 1998) of stress. Habituation to prolonged exposure to stressful events could possibly explain why tinnitus patients do not report any changes after the stress test. The lack of cortisol differences is in line with the findings of several other reports documenting no differences in endocrine responses to chronic stress (Hamilos et al., 1998; Scott and Dinan, 1998; Gaab et al., 2001).

In addition, while sAA levels were lower in the subjects with tinnitus, no changes could be obtained between subjects with tinnitus and healthy controls throughout the experiment for salivary cortisol. These findings further suggest dissociation between the two stress systems (endocrine vs. autonomic), and further point to a specific nature of the SNS-HPA axis relationship. Recent research already suggested an asymmetry between the HPA axis and SNS (Nater and Rohleder, 2009). The factors responsible for this asymmetry are largely unknown. One possible explanation could be that when it comes to habituation, these two systems operate at different rates. In support of this idea, evidence has been provided that in experimental animals, repeated stress exposure leads to a decreased response over time in the SNS with sustained HPA axis response (Britton et al., 1992). Whatever the causes, it has been suggested that asymmetry between these two biological systems may have unhealthy consequences (Bauer et al., 2002) and might contribute to pathogenesis development and/or maintenance (Monteleone et al., 2011).

Research has suggested that neopterin production might be linked with immune pathogenesis (Zhang et al., 2009; Chittiprol et al., 2010). It is known that neopterin is a significant and reliable indicator for the endogenous formation of IFN- γ (Fuchs et al., 2009). A prolonged elevation of IFN-γ was suggested to induce hyperexcitability neurons, which may lead to amplify pain perceptions, a process referred to as central sensitization (Vikman et al., 2007). Interestingly, tinnitus has also been considered a central sensitization phenomenon that results from an increase in responsivity to neural activity (Zenner, 2006; Zenner et al., 2006). Overall, numerous studies have shown an association between increase in immune system function due to infections (Fuchs et al., 1988, 1993b; Avanzas et al., 2004; Breinekova et al., 2007; Chittiprol et al., 2010; Euteneuer et al., 2012) and deregulation of endocrine and autonomic systems. However, we were unable to make such an assumption because in this study subjects were excluded if they had infectious or inflammatory diseases. On the other hand, our results do support variability in salivary neopterin that seems to be independent of fluctuation in sAA and salivary cortisol, suggesting that there also an asymmetry with neopterin. However, we are not aware of any former study that reports on the exact relationship between salivary measures of neopterin and respectively sAA or salivary cortisol; therefore, further examination of this assumption is necessary.

### Feasibility of using salivary stress-related biomarkers

sAA levels in this study showed statistically significant lower values in the subjects with tinnitus compared to subjects without tinnitus and sAA levels were correlated with stress. After correction for salivary cortisol and neopterin levels, the effects remained significant. As such, these results suggest a promising role of sAA as a possible biological stress marker in tinnitus. Given that in subjects without tinnitus, sAA was able to measure acute stress, it suggests that subjects with tinnitus had an impaired or inadequate functioning of the SNS.

## Limitations

Although the present study has yielded significant findings in regard to the use of stress-related markers—specifically with regard to salivary α amylase—its design is not without limitations. Thus, a number of caveats need to be noted regarding the present work. First, this is a preliminary study, and thus it was primarily limited by its small sample size and statistical need to run multiple comparisons. Second, ideally including subjects of both sexes and increasing the collection range to longer time period would expand the sample size and further aid in generalizing the findings. Third, the small number of subjects may have led to an underestimation of the differences in salivary markers. Fourth, due to time constraints only a portion of the TSST test was administered, and although the portion administered is proven to induce stress, administering the entire TSST would have probably led to a larger increase in stress. Finally, although there was no significant rise in salivary cortisol secretions after the stressful event, subjective reports indicated that the stress-induced task used was effective enough to induce stress as measured by salivary α amylase.

## Conclusion

Although stress can be a versatile event, reliable biological indicators of stress reactions have been shown to be valuable markers in both psycho physiological research and clinical practice. As such, alteration in the psychological state of a patient with tinnitus can alter biological indictors of stress reactions as measured by salivary stress related biomarkers. With this in mind, stress related markers have been emerged as potential non-invasive techniques with collection methods that can be administrated by health and non-health professionals. As a final point, it is important to note that even if salivary measures of stress do not map well due to their sensitivity, they may yet predictive of diseases and well-being. Future research relating the validity of using salivary stress related measures is needed to better determine their clinical relevance. Particularly, studies should further confirm whether sAA is a sensitive maker of stress and how it is associated with other mediators of the allostatic load network, such as pro- and anti-inflammatory cytokines (e.g., interleukin-6, tumor necrosis factor-alpha).

## Author contributions

OA collected and analyzed the data, prepared and wrote the manuscript, as well as secured the funding. DT monitored data collection, advised on data analyses, advised on manuscript perpetration. SV data analyses, manuscript perpetration, and manuscript writing.

## Funding

This work was supported by the American Tinnitus Association (ATA, funding #: 225352).

### Conflict of interest statement

The authors declare that the research was conducted in the absence of any commercial or financial relationships that could be construed as a potential conflict of interest.
